# Creating a responsible authorship culture in science: Anchoring authorship practices in principles of transparency, credit, and accountability

**DOI:** 10.1073/pnas.2531268123

**Published:** 2026-03-11

**Authors:** Véronique Kiermer, Sofia Adams, Kirsten Bibbins-Domingo, Yensi Flores Bueso, Kathleen Hall Jamieson, Joerg Heber, Mohammad Hosseini, Ana Marušić, Beau Nielsen, Magdalena Skipper, Geeta K. Swamy, Susan M. Wolf

**Affiliations:** ^a^Public Library of Science, San Francisco, CA 94103; ^b^Annenberg Public Policy Center, University of Pennsylvania, Philadelphia, PA 19104; ^c^Journal of the American Medical Association and the Journal of the American Medical Association Network, Chicago, IL 60611; ^d^Cancer Research, University College Cork, Cork T12 XF98, Ireland; ^e^Institute for Protein Design, University of Washington, Seattle, WA 98195; ^f^Annenberg School for Communication, University of Pennsylvania, Philadelphia, PA 19104; ^g^Lawrence Berkeley National Laboratory, Berkeley, CA 94720; ^h^Department of Preventive Medicine, Northwestern University Feinberg School of Medicine, Chicago, IL 60611; ^i^Department of Research in Biomedicine and Health and Center for Evidence-based Medicine, University of Split School of Medicine, Split 21000, Croatia; ^j^National Academies of Sciences, Engineering, and Medicine, Washington, DC 20001; ^k^Nature, London N1 9XW, United Kingdom; ^l^Department of Obstetrics & Gynecology, Duke University School of Medicine, Durham, NC 27710; ^m^University of Minnesota Law School and Department of Medicine, University of Minnesota Medical School, Minneapolis, MN 55455

**Keywords:** authorship, accountability, transparency, credit

## Abstract

Authorship remains the primary currency of academic credit and a cornerstone of research integrity, yet current practices often fail to reflect the collaborative and interdisciplinary nature of modern science and questionable authorship practices persist. We argue that addressing these shortcomings is a collective responsibility shared by researchers, journals, research funders, scholarly societies, and research institutions. We examined authorship guidelines issued by journals and research institutions and found that their recommendations to researchers are highly variable. We propose that fostering a responsible authorship culture requires a shared, principle-based framework grounded in transparency, credit, and accountability. These three interconnected principles highlight when authorship practices are questionable and offer a framework for constructive reflection on the meaning of authorship. We outline practical ways research leaders can embed these principles into everyday practice by initiating early, inclusive, and fair authorship discussions and ensuring transparent description of contributions. Research institutions have a unique opportunity to inculcate good practices and lead this culture change with harmonized guidance, education, fair conflict resolution, and reform of researcher assessment. Anchoring authorship in transparency, credit, and accountability will strengthen the credibility of individual research, the fairness of recognition systems, and, ultimately, the trustworthiness of science itself.

In his 1969 essay “What is an Author?,” Michel Foucault, quoting the playwright Samuel Beckett, asked “What does it matter who is speaking?” (1) In the case of scientific publication, the answer is, it matters a lot—for individuals, for the scientific community, and for science to be trusted. For individual researchers, authorship remains a central unit of academic recognition, reward, and career progression. For the scientific community at large, honest and transparent authorship practices protect the integrity of research findings (2). For science itself, inclusive authorship that reflects a collaborative design of research can increase trust and perceived relevance of results. Yet, authorship disagreements and questionable practices remain a common concern for researchers (3), university administrators (4), research funders (5, 6), and journal editors (7). Despite well-publicized authorship guidelines (8, 9), debates about whose contributions are worthy of authorship are frequent, complex, and high-stakes.

Because creating a responsible authorship culture that reduces these conflicts is a cornerstone of an effective and fair research enterprise, it is the collective responsibility of the entire scholarly community—including researchers at all levels of seniority, journals, research funders, scholarly societies, and research institutions. In an examination of authorship guidelines issued by two of these groups—journals and research institutions—we found that their recommendations to researchers are highly variable. We argue that harmonization of guidelines can help but also that guidelines alone are insufficient to promote culture change. A responsible authorship culture requires a principle-based reflection by research teams on what it means to be an author with support and encouragement from research institutions. Accordingly, we propose three interconnected principles that form the foundation for responsible authorship: transparency, credit, and accountability (Fig. 1).

**Fig. 1.**
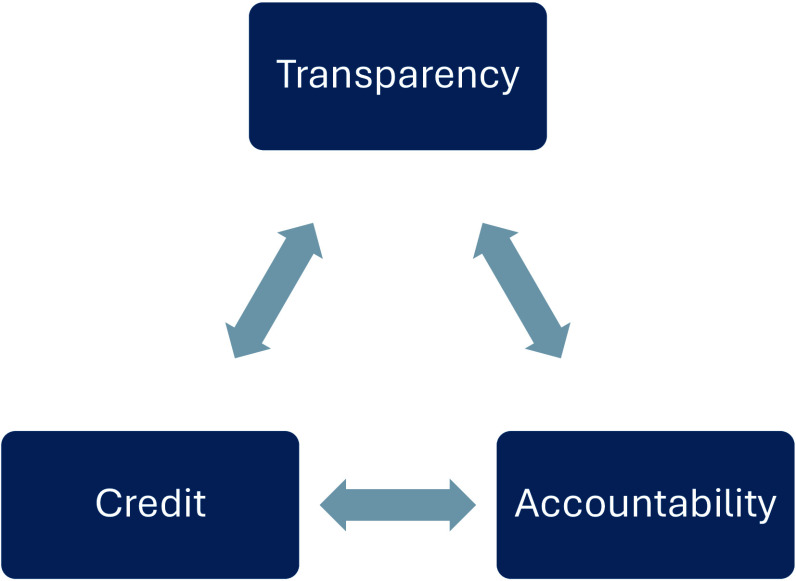
Three principles forming the foundation for responsible authorship.

We also offer three ways in which research team leaders can embed best practices for transparency, credit, and accountability in their ongoing work: i) anchor authorship decisions in the principles of transparency, credit, and accountability that underlie authorship guidelines; ii) establish a fair, robust, and inclusive process to make authorship decisions, addressing authorship criteria early and revisiting them as frequently as necessary; and iii) transparently describe all authors’ contributions (see Box 1). We argue that universities and other institutions conducting research have a unique opportunity to lead the promotion of a responsible authorship culture and are uniquely positioned to do so by supporting the adoption of these recommendations. In what follows, we explain the rationale for the three recommendations. We discuss the role research institutions can play in their adoption, and highlight why they should be supported in this effort by the research ecosystem.

Box 1. Authorship principles in action.

**Figure:**
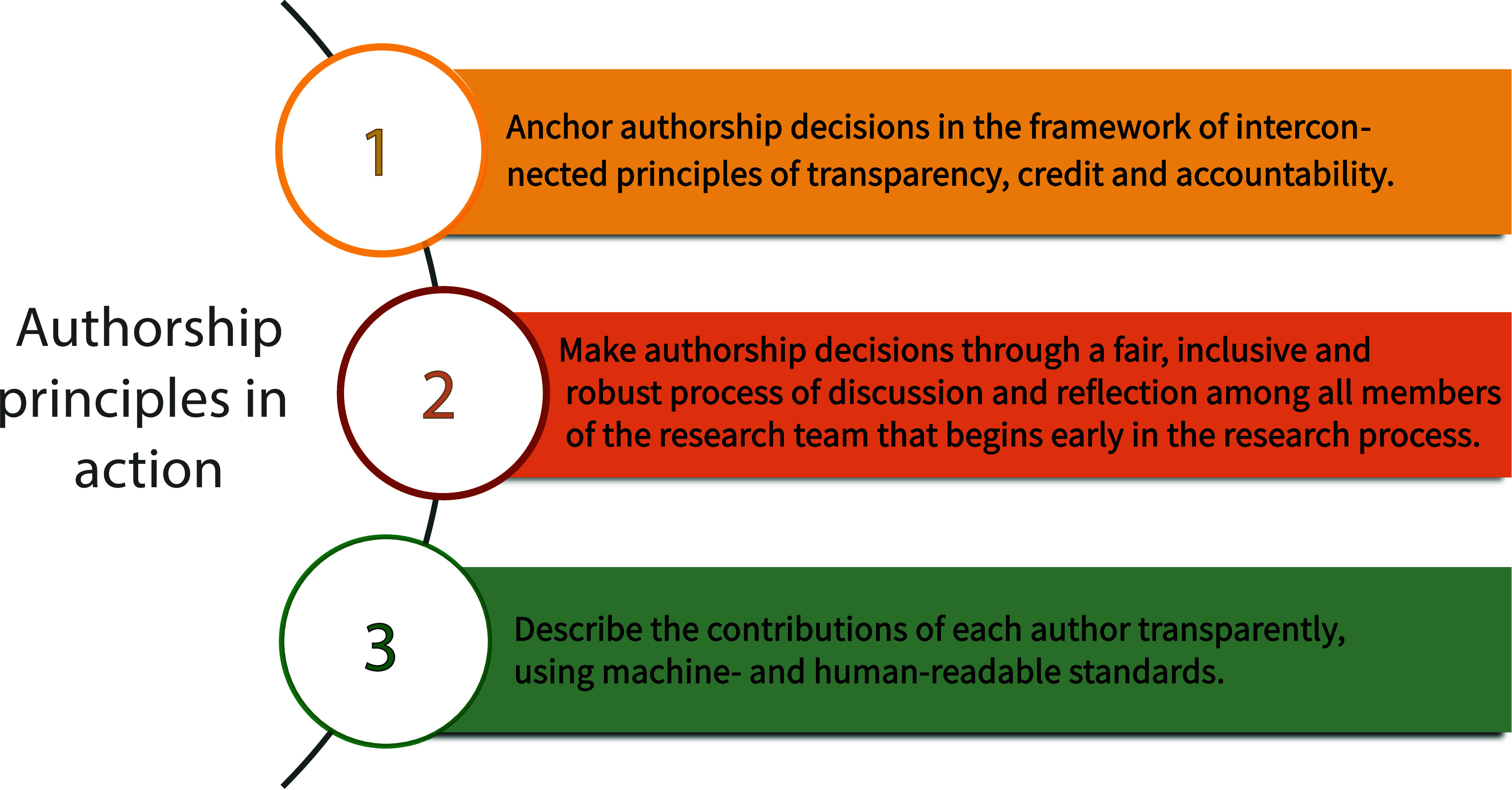

## Authorship Practice Must Evolve with Research Practice

The 2017 National Academies consensus report, *Fostering Integrity in Research* (2), observes, “The challenges that the research enterprise faces in the area of authorship are exacerbated by a tension between the conventions of authorship, which assume a unitary authority who can vouch for the entirety of the work, will receive most of the credit for it, and decide who else will be recognized and how, and the way a significant fraction of research activity is actually undertaken today.” Research increasingly takes place in multidisciplinary teams and in collaboration with contributors outside of academia. In these circumstances, some of the older authorship conventions become untenable. For example, every author cannot always realistically be accountable for all aspects of the work and every author may not necessarily have participated in drafting the article or have the expertise to critically review the entire manuscript. The report’s authors urge the research enterprise to accelerate progress in authorship standards and thus “make a significant contribution to research integrity.” Consistent with that realization, a more recent National Academies consensus report, *The Science and Practice of Team Science* (10), calls for funders and research institutions to support team science with interventions spanning the entire life cycle of a team science project, from development through reporting. It also urges journals to adopt “clear policies and guidelines for authorship, allocation of credit, and contribution level, particularly when there are many contributing authors and when authors are contributing from different disciplines with different authorship practices.”

We examined a selection of journal authorship guidelines and found considerable variability in how the journals formulate them (Dataset S1). The journals examined included primarily those represented in a group convened by the National Academies in 2018 who issued recommendations on authorship (9). At the time of our survey in May 2025, only a subset of the journals surveyed had adopted these 2018 recommendations. Notably, many journals, across a broad range of disciplines, referred to the guidelines of the International Committee of Medical Journal Editors (ICMJE) (8). Other journals offered fewer details or customized language.

The call for standards and for specificity is understandable since empirical studies have shown a great degree of variation in how researchers perceive and understand existing authorship criteria and how they apply them in practice (3, 11, 12). Discipline-specific conventions also create wide differences in how the guidelines are applied. For example, author ordering that is often alphabetical in economics implies the degrees of contribution in biology. Considering how deeply these norms are entrenched in academic culture and the variations in how research is conducted, we argue that to establish a reasonable set of specific criteria that could universally apply to all possible authorship situations is an impossible task. The outcome would also be short-lasting: The inadequacy of current criteria to effectively support team science is one example of how the evolution of research practices challenges conventional approaches. As useful as efforts to increase specificity may be, they will inevitably apply to a subset of circumstances. To complement these efforts, we advocate foregrounding principles able to transcend situational and disciplinary differences.

## The Inseparable Principles of Credit and Accountability

While authorship guidelines vary in level of detail and specificity, the major ones build on expectations of both credit and accountability for authors. The authorship recommendations of the ICMJE (8), the Committee on Publication Ethics (COPE) (7), and major scientific publishers^*^ all recognize, as Rennie and Flanagin (13) observed, that “the coin [of academic credit] has two sides, and the reverse is responsibility” (13).^†^ By becoming an author of any research output—a conference paper, dataset, code, preprint, or journal article—contributors not only assert that they deserve to be recognized as authors for what they did but also assume responsibility for that work and hence accountability for its integrity. In short, credit entails accountability and accountability entails credit—the two are inseparable.

**Fig. 2.**
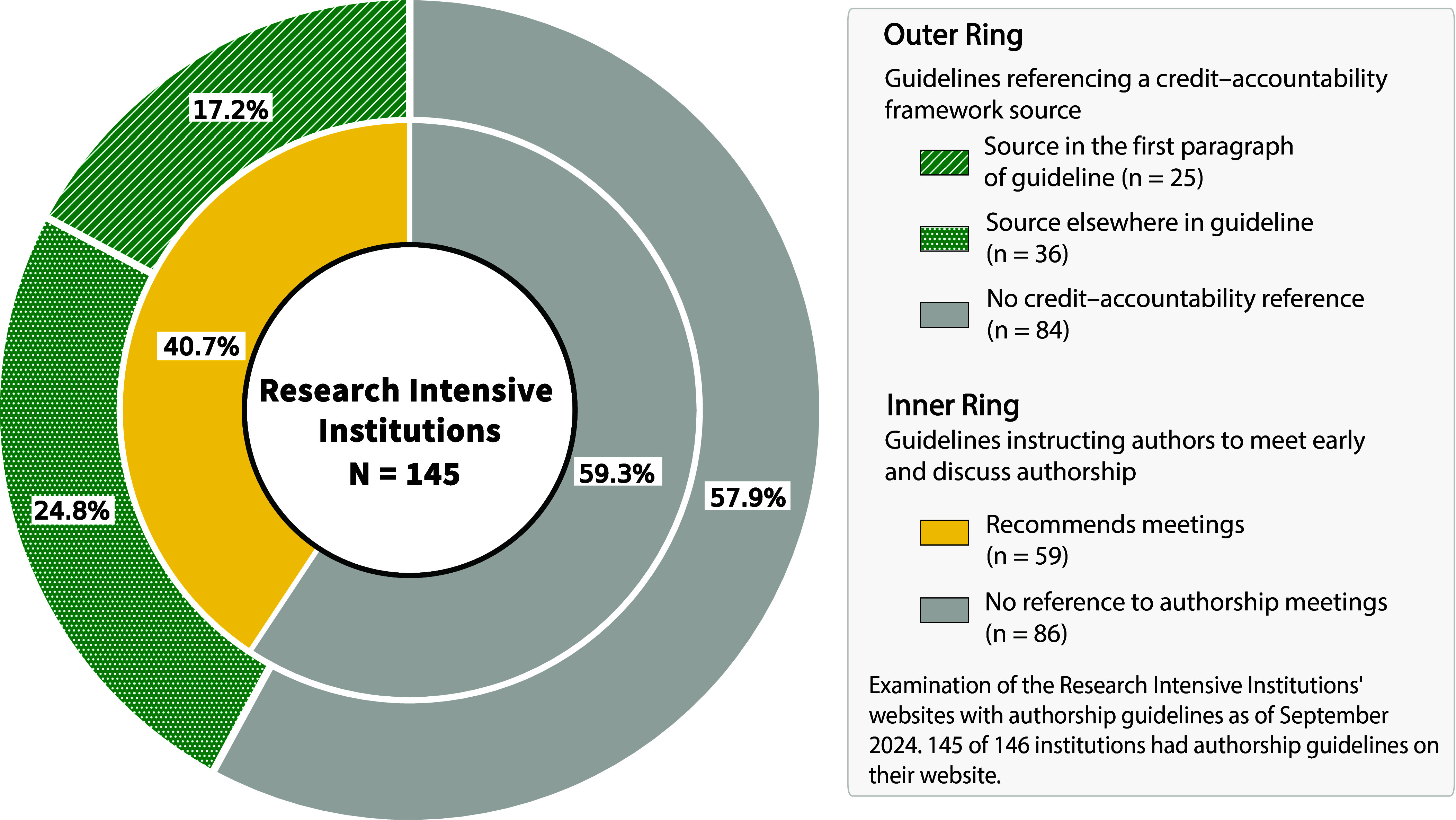
As of September 2024, examination of 145 US Research Intensive Institutions’ websites. Those counted as associating credit with accountability did so through a mention of this relationship in the text of the authorship guidelines posted on their external-facing website. Those that linked to external guidelines (e.g., ICMJE, AAAS, COPE, Nature Portfolio) but did not otherwise mention the association explicitly were not counted.

The credit-accountability lens is evident in some, but not most, of the authorship guidelines posted on the websites of major academic research institutions in the United States. An Annenberg Public Policy Center search conducted in September 2024 located authorship guidelines of some sort on the websites of 145 of the 146 R1 US universities (14). Of those 145, a total of 61 (42%) explicitly associated credit with accountability at some point in the document, and 25 (17%) contextualized authorship expectations by featuring the credit-accountability frame in the first paragraph (Fig. 2). A small proportion of guidelines on university websites also linked to other authorship guidelines: 6 of the 145 university authorship statements linked to the ICMJE statement; 1 to the authorship statement by the American Association for the Advancement of Science (AAAS) that links in turn to the ICMJE statement; 1 to the COPE statement; and 1 to the Nature Portfolio statement, which is based on the 2018 recommendations by a group convened by the National Academies (9).

Combined with transparency, the inseparable principles of credit and accountability form a framework that clarifies what is problematic about questionable authorship practices—they tend to emphasize credit without accountability and thus threaten the integrity of research. The 2017 National Academies report identified three detrimental research practices that impair the fundamental function of authorship to support integrity: gift or honorific authorship, ghost authorship, and coercive authorship (2). Under the transparency-credit-accountability framework, a person is deserving of, and entitled to, authorship only if they can be held accountable for defects in the work, should they arise, or if disclosure of their identity would reveal biases and forms of self-interest that might affect the work’s approach, interpretations, or conclusions. A named author who has not contributed to the work cannot be accountable for the actual research and hence, deserves no credit. This is true for honorific authors as well as individuals who make coercive demands for authorship—e.g., demands solely based on seniority within the group or as a condition of access to a resource that they control while it should be considered a public good. Conversely, a person who should be held accountable for the reliability of the work assumes responsibility and hence merits inclusion and credit. Ghost authorship is unacceptable because suppressing the identity of a person who played a role in the work makes it impossible to hold that person accountable not only for any defects in the work but also for disclosing potential biases or conflicts.

## Recommendation 1: Anchor Authorship Decisions in Transparency, Credit, and Accountability

While the principles of transparency, credit, and accountability underpinning authorship guidelines are robust, the way authorship criteria are typically formulated (in universities and journal guidelines) is often too inflexible to adjust to new ways in which research is conducted. For example, in large asynchronous multisite international collaborations, in multidisciplinary teams, and in research teams including individuals outside of academia, some team members may have met only some of the traditional authorship criteria (7, 15). They may have made substantial contributions to the acquisition of data, or the development of code for its analysis, but may not have drafted or critically reviewed the work. In these circumstances, it is also often unrealistic to hold every author accountable for all aspects of the work. These limitations may be due to lack of specialized knowledge or specific expertise. Yet, all authors who deserve credit must remain accountable for their own contributions. And at least some authors must take responsibility to ensure that questions related to the accuracy or integrity of any part of the work, even ones in which the author was not personally involved, are appropriately investigated, resolved, and the resolution documented.

Addressing the challenges related to the rapid evolution of the research environment and workforce starts with transparency, ensuring that each team member’s contributions are clearly described. Then, anchoring the attribution of authorship in the inseparable principles of credit and accountability offers a way forward. Although the transparency-credit-accountability framework does not provide a formula, it encourages reflection and a starting point for discussing the importance of the diverse skills, know-how, and experience of individual researchers for the integrity, appropriateness, and relevance of the work.

Such a principle-based discussion, in contrast to merely a criteria-ticking exercise, also opens space for reflection on accountability, and specifically to whom the authors should be accountable. For example, research teams increasingly ensure their accountability to the scientific community (and to their funders) by adopting open science practices and sharing intermediary outputs—data, code, methods—that others can examine, question, reproduce, and apply in other studies. Because making such uses possible is both effort-intensive and helps ensure that research is replicable and reproducible, those who perform this function should be considered for authorship in a transparency-credit-accountability framework (16).

Researchers are also accountable to the populations they study. Increasingly, journals demand better representation of the perspectives of the studied communities in the research process, which can lead not only to richer, more nuanced results but also to ones more likely to be trusted by these populations.^‡^ Where this is considered essential, credit may be justified for the community as a group [through group authorship (17)] or for its representatives who ensure that the research is informed by community perspectives and reflects community values and needs.

The interconnected principles of transparency, credit, and accountability also help address emerging challenges. For example, in the past few years, debates have emerged on whether AI tools could be considered authors of research articles. In our survey of journals, those that had a policy stated that AI tools cannot be authors because they cannot fulfill the principle of accountability. Journals that allow the use of AI tools require disclosure of how these tools have been used—in line with the transparency principle. The prevailing opinion remains that authorship practices should be tied to all three principles of transparency, credit, and accountability.

Concrete recommendations include:

- Research institutions, journals, funders, and scholarly societies should adopt publicly posted guidelines expressly linking authorship to credit due for contributions to the published work plus accountability for the quality and integrity of one’s contributions and their representation in the work.
- Authorship guidelines should recognize that in modern, multidisciplinary research, it may be unrealistic to expect all authors to take responsibility for the full work. However, accountability requires that at least one team member is identified as responsible for the quality and integrity of the published work. As ICMJE recommends (8), this entails the ability to identify which coauthors are responsible for specific parts of the work.

## Recommendation 2: A Fair and Robust Process

The transparency-credit-accountability principles do not explicitly address all situations, but they provide a framework for discussion and decision-making. It is important that these discussions follow a fair process. Cooke et al. offer 10 strategies for these conversations (18), including initiating authorship discussions early, documenting contributions transparently, and communicating frequently about any deviations from the original plan. However, the Annenberg Public Policy Center’s analysis of authorship guidelines on R1 universities’ websites found that only 59 out of 145 (41%) instructed prospective authors to meet to discuss authorship expectations (Fig. 2).

The strategies recommended by Cooke et al. also stress that a responsible process should be inclusive and mindful of power dynamics and implicit biases. For example, all members of the research team should be encouraged to discuss authorship and author order (in disciplines where it matters), based on the transparency-credit-accountability framework. This will ensure transparency throughout and should be a routine part of conducting the research and progressing to publication. A junior researcher should be able to challenge the interpretation by a senior team member of the data the junior researcher contributed, with both of them contributing to the publication’s presentation of the results. Inclusive dialogue provides opportunities for junior and senior contributors to make and assess contributions in light of the transparency-credit-accountability framework. Similarly, these value-based discussions should help address and correct any biases against marginalized groups. As Cooke et al. stress, “Coauthorship conversations are critically important when collaborating with groups that are chronically underrepresented or even exploited in science (e.g., Indigenous knowledge holders).”

With research increasingly funded by the private sector and the rise of citizen science, more research will be published by non-university-affiliated researchers or by collaborative groups crossing academia and industry or other private entities. To promote an open and socially engaged research culture, with increased participation and representation of citizen scientists and other groups outside academia, is important to bolster trust in the research process. A robust authorship process, thoughtfully considering power dynamics and competing interests is equally important (15, 19). The recommendations provided here apply equally to this type of research.

Concrete recommendations include:

- Research institutions, journals, funders, and scholarly societies should do more than post guidelines on authorship. They should urge that all members of a research team discuss from the start of a collaboration who will receive authorship credit, anticipated author order, and other forms of expected credit (such as recognition in the paper’s Acknowledgments). The team should record the plan to ensure transparency and revisit it periodically to reconfirm or make adjustments.
- Ensuring the active participation of junior contributors in these discussions from the start is crucial to support proper credit and accountability. Involving the full team, including junior and senior contributors, can reduce misunderstanding and conflict, model responsible authorship practices, and increase the likelihood that team members will initiate these practices throughout their career.

## Recommendation 3: Transparent Description of Contributions

The scientific community has an opportunity to make the transparency-credit-accountability framework a living set of practices based in a strong culture integrity. Increasingly, journals require author contribution statements describing the contributions made by each author and tools are now available to support the description of contributions. For example, the CRediT taxonomy is a human- and machine-readable list of standard contributor roles (20). Importantly, CRediT does not determine authorship but instead describes the contributions of individuals. Having a consistent vocabulary can facilitate a transparent and fair process for authorship discussions (21) and subsequently, describing all authors’ contributions in standardized ways helps enhance their visibility and discoverability (22). When new research practices make particular contributions important for accountability, transparent descriptions can signal new patterns which in turn can reinforce emerging discipline norms. Transparency can also facilitate investigation when error or fraud is suspected. The CRediT taxonomy requires continued stewardship to accurately reflect the evolving diversity of contributions, and its usefulness can still be improved (23, 24). We recommend the continued use and development of the CRediT taxonomy because it lays the foundation for the transparency element of the transparency-credit-accountability framework that supports scientific integrity (9).

When stressing the importance of the link between credit and responsibility in 1997 (25), Rennie et al. also proposed replacing what they saw as the constrictive notion of authorship with the more capacious “contributorship,” a concept that invites detailed, transparent disclosure of the part everyone played in the research, documenting, and writing process. They argued that such radical change is necessary “to reflect the realities of multiple authorship and to buttress accountability.” This call for a move to contributorship has been repeated many times either as a complete replacement for authorship (for example, see refs. 7 and 26–28) or as the addition of a “nonauthor contributors” category named in acknowledgments (8). Both suggestions, however, face practical obstacles in the publishing and research assessment systems: There is currently no robust mechanism for ensuring that research assessment committees consider contributors, and contributors are deprived of downstream credit. To break the link between accountability and credit is to risk unintended consequences that undermine the culture of responsible authorship. However, a central motivation of the more granular contributorship model was to include both conceptual and technical contributions. In a responsible authorship model, the transparency-credit-accountability principles imply the recognition of all contributions important for accountability.

Concrete recommendations include:

- The corresponding author should carefully describe all contributions to the work when submitting it for publication and specifying the authors. The CRediT taxonomy remains a useful and standardized approach to describe contributions. All journals should implement it or emulate it with human- and machine-readable taxonomies to describe all types of research contributions that require accountability.
- Research practices evolve over time. The CRediT taxonomy helps describe contributions but does not determine which contributions qualify for authorship. The standards committee stewarding CRediT (and the authorities maintaining similar taxonomies) should review the taxonomy regularly to ensure it reflects all important contributions that are crucial to upholding the principles of credit, accountability, and transparency (24). Journals should adopt mechanisms to consider requests to recognize new contributions warranting credit and accountability.

## Building a Responsible Authorship Culture

An authorship culture based on transparency, credit, and accountability depends on fostering institutional cultures that live these principles. This should be a priority for the entire research ecosystem and supported by journals, research funders, and disciplinary societies. The responsibility for creating and sustaining this culture is shared, and research institutions have opportunities to develop specific interventions—small and large—to lead this cultural change, which should be supported by all actors.

First, universities and other institutions conducting research can provide better guidance. As the Annenberg Public Policy Center’s analysis of R1 universities shows, authorship guidelines on institutions’ websites are variable but tend to focus on specific criteria instead of integrating principles. Research institutions have an opportunity to align their guidelines and telegraph the importance of the transparency, credit, and accountability framework. The same recommendation applies to journals, research funders, and scholarly societies.

Second, institutions can support implementation of this framework by providing educational material and courses that promote a responsible authorship culture and by setting expectations for robust, fair, and inclusive authorship decision processes as well as transparency of contributions, as an integral aspect of conducting research (5). These efforts can be further supported by journals, research funders, and scholarly societies by developing additional educational material.

Third, supporting implementation also means that institutions need to play an active role in supporting research laboratories, groups, and leaders in appropriately addressing authorship issues and, if these mechanisms fail, providing institutional means to resolve the conflict. Institutional policies and guidelines can reaffirm the inseparable principles of transparency, credit, and accountability; articulate principles for dispute resolution, including points of contact and the resolution process; and finally establish retaliation protections for research contributors voicing their concerns (see, e.g., ref. 29). Institutional policies can strive to provide a trusted and neutral environment that minimizes conflicts of interest and mitigates concerns about power differentials.

Fourth, any culture change requires reinforcement and institutions hold the key to a major reinforcement mechanism—the assessment of researchers during hiring, promotion, and annual reviews. Globally, the assessment of research performance in academia relies heavily on publication outputs, with some regional and context variation (30). Very much like our call for improved authorship culture and processes, a number of initiatives call for revised research assessment frameworks that align with evolving research practices, embed principles of rigor and accountability, and safeguard integrity. These initiatives include the Coalition and Agreement on Reforming Research Assessment [CoARA, 2022 (31)], the Declaration of principles on research assessment of the Latin American Forum for Research Assessment, Latin American Council of Social Sciences [CLACSO-FOLEC, 2020 (32)], the Hong Kong Principles for assessing researchers [2020 (33)], the Leiden Manifesto for research metrics [2015 (34)], and the Declaration on Research Assessment [DORA, 2013 (35)]. These initiatives call for recognizing the full range of scientific contributions and valuing diverse outputs such as datasets, software, methods, policy inputs, and community engagement. They advocate for inclusivity and social responsiveness, so that research addresses local and regional needs while promoting equitable recognition across languages and contexts. Importantly, they highlight the role of team science, acknowledging the diverse expertise required for modern research. The recent National Academies consensus report, *The Science and Practice of Team Science*, provides specific recommendations related to researcher assessment by institutions and research funders (10). Institutions have an important opportunity to align their assessment framework—during hiring, annual reviews, tenure, and promotion—and their authorship culture, and to make this link clear in evaluation documentation and practice, in order to create mutually reinforcing mechanisms for a strong and responsible research culture. Similarly, research funders have the opportunity to further that alignment in their grant review process.

Fifth, assessment is crucial to evaluating the impact of these changes. Research institutions and journals can take the lead in collecting quantitative and qualitative data to gauge success and make needed adjustments. Content analysis of posted guidelines, anonymous surveys to assess behavior and satisfaction, longitudinal analysis of trends in authorship disputes, and examining assessment frameworks are among the tools available for building a data-informed system to guide progress. Especially powerful would be agreement on consensus measures that would allow individual institutions and journals to measure their own progress while revealing broader aggregate trends.

Box 2. Opportunities to foster a responsible authorship culture.

**Table:** 

Interventions	Research Institutions	Journals	Research Funders	Scholarly Societies
Clarify authorship guidance to foreground the principles of transparency, credit, and accountability	✔	✔	✔	✔
Provide training and set expectations for trainees and faculty to jointly practice a robust authorship decision process	✔	✔	✔	✔
Establish a robust and fair conflict resolution process	✔			
Reinforce transparency, credit, and accountability principles in researchers’ assessment.	✔		✔	
Collect quantitative and qualitative data to evaluate the impact of these reforms and support data-informed progress.	✔	✔		

## A Responsible Enterprise Effort

Authorship and publication of research articles remain fundamental to academic recognition and reward. A trustworthy system requires that the credit and accountability discussions start early, when the research is conceived, and evolve transparently as the research is conducted. Authorship formalizes credit and accountability and should faithfully and transparently reflect the research process. Institutions conducting research have an opportunity and responsibility to inculcate good practices and lead in creating a responsible authorship culture. All who influence incentives and oversight in science should join in this effort: journals, scholarly societies, institutions such as the National Academies, and research funders. A responsible authorship culture is the cornerstone of a responsible research enterprise.

## Supplementary Material

Appendix 01 (PDF)

Dataset S01 (XLSX)

## Data Availability

All data are included in the manuscript and/or supporting information.

## Footnotes

^*^For example, several journal families have adopted a derivative of ICMJE recommended in reference 9: Nature Portfolio, “Authorship.” [Accessed January 8, 2026.] https://www.nature.com/nature-portfolio/editorial-policies/authorship; Science Portfolio, “Authorship” [Accessed January 8, 2026.] https://www.science.org/content/page/science-journals-editorial-policies#authorship; PLOS Portfolio, “Authorship Requirements.” [Accessed January 8, 2026.] https://journals.plos.org/plosone/s/authorship#loc-authorship-requirements. The publisher Wiley has adopted both ICMJE and American Psychological Association (APA) recommendations. https://www.apa.org/pubs/journals/resources/publishing-tips/giving-credit.

^†^The ICMJE’s recommendations on “Defining the Role of Authors and Contributors” open by declaring that “Authorship confers credit and has important academic, social, and financial implications. Authorship also implies responsibility and accountability for published work.” The first sentence under “Authorship” in the Nature Portfolio recommendations declares that “Authorship provides credit for a researcher’s contributions to a study and carries accountability.” And the opening paragraph of the COPE “Protecting Integrity in Research and its Publication” states that “Authorship conveys significant privileges, responsibilities, and legal rights; in the scholarly arena, it also forms the basis for rewards and career advancement.”

^‡^Several journal groups have introduced policies on inclusivity in global research (and address the issue known as ‘parachute science’), for example: PLOS (https://journals.plos.org/plosone/s/best-practices-in-research-reporting#loc-inclusivity-in-global-research), Nature (https://www.nature.com/nature/editorial-policies/authorship#authorship-inclusion-and-ethics-in-global-research), AGU (https://www.agu.org/publications/authors/policies#authorship), and eLife (https://elifesciences.org/inside-elife/5f07c933/author-policy-elife-addresses-parachute-science-to-support-meaningful-collaborations). Another example is The BMJ’s policies on patient and public partnership (https://www.bmj.com/campaign/patient-partnership#). [Accessed August 25 2025].
