# Supplementary material for: Creating a responsible authorship culture in science: Anchoring authorship practices in principles of transparency, credit, and accountability
Source: Proc Natl Acad Sci U S A. 2026 Mar 11;123(12):e2531268123. doi: 10.1073/pnas.2531268123 (PMC13012122; doi:10.1073/pnas.2531268123)
Supplement: Supplementary file 1 — Appendix 01 (PDF) [file pnas.2531268123.sapp.pdf]

# Supporting Information for

Creating a responsible authorship culture in science: Anchoring authorship practices in principles of transparency, credit, and accountability.

Véronique Kiermer[1,2,3], Sofia Adams[4], Kirsten Bibbins-Domingo[5], Yensi Flores Bueso[6], Kathleen Hall Jamieson[7,3], Joerg Heber[8], Mohammad Hosseini[9], Ana Marušić[10], Beau Nielsen[11], Magdalena Skipper[3,12], Geeta K. Swamy[13], Susan M. Wolf[3,14]

Corresponding author:
Véronique Kiermer
vkiermer@plos.org

**This PDF file includes:**

Legend for Dataset S1

[1] To whom correspondence may be addressed. Email: vkiermer@plos.org.
[2] Public Library of Science, San Francisco, CA 94111. https://orcid.org/0000-0001-8771-7239
[3] Members of the Strategic Council for Research Excellence, Integrity, and Trust.
[4] Annenberg Public Policy Center, University of Pennsylvania, Philadelphia, PA 19104. https://orcid.org/0009-0000-7549-2749
[5] JAMA and the JAMA Network, Chicago, IL 60611. https://orcid.org/0000-0002-8962-0622
[6] University College Cork, Cork T12YT20, Ireland and University of Washington, Seattle, WA 98195. https://orcid.org/0000-0002-2118-2195
[7] Annenberg Public Policy Center, University of Pennsylvania, Philadelphia, PA 19104. https://orcid.org/0000-0002-4167-3688
[8] Lawrence Berkeley National Laboratory, Berkeley, CA 94720. https://orcid.org/0000-0002-6370-4254
[9] Northwestern University Feinberg School of Medicine, Chicago, IL 60611. https://orcid.org/0000-0002-2385-985X
[10] University of Split School of Medicine, Split, Croatia. https://orcid.org/0000-0001-6272-0917
[11] Staff of the Strategic Council for Research Excellence, Integrity, and Trust. National Academies of Sciences, Engineering, and Medicine, Washington, DC 20001.
[12] Nature, London N1 9XW, United Kingdom. https://orcid.org/0000-0001-8707-8369
[13] Duke University, Durham, NC 27710. https://orcid.org/0000-0001-5092-6993
[14] University of Minnesota Law School and Medical School, Minneapolis, MN 55455. https://orcid.org/0000-0001-9547-2200

**Dataset S1. Survey of a sample of journal authorship guidelines.** The journals examined included primarily those represented in a group convened by the National Academies in 2018 who issued recommendations on authorship (reference 9; McNutt et al., 2018). The authorship guidelines were accessed on May 9, 2025 (with some updates noted on August 7, 2025). The survey examined to what extent the journal families have adopted the McNutt et al, 2018 recommendations among other characteristics.
